# Defining the Criteria for Reflex Testing for *BRAF* Mutations in Cutaneous Melanoma Patients

**DOI:** 10.3390/cancers13092282

**Published:** 2021-05-10

**Authors:** Sarah Zhou, Daniel Sikorski, Honghao Xu, Andrei Zubarev, May Chergui, François Lagacé, Wilson H. Miller, Margaret Redpath, Stephanie Ghazal, Marcus O. Butler, Teresa M. Petrella, Joël Claveau, Carolyn Nessim, Thomas G. Salopek, Robert Gniadecki, Ivan V. Litvinov

**Affiliations:** 1 Division of Dermatology, McGill University, Montreal, QC H3A 0G4, Canada; sarah.zhou@mail.mcgill.ca (S.Z.); daniel.sikorski@mail.mcgill.ca (D.S.); awz.research@gmail.com (A.Z.); francois.lagace@mail.mcgill.ca (F.L.); stephanie.ghazal@mail.mcgill.ca (S.G.); 2 Division of Dermatology, Laval University, Quebec City, QC G1V 0A6, Canada; hong-hao.xu.1@ulaval.ca (H.X.); joel.claveau@videotron.ca (J.C.); 3 Department of Pathology, McGill University, Montreal, QC H3A 0G4, Canada; May.Chergui@MUHC.MCGILL.CA (M.C.); margaret.redpath@mail.mcgill.ca (M.R.); 4 Departments of Medicine and Oncology, McGill University, Montreal, QC H3A 0G4, Canada; wilson.miller@mcgill.ca; 5 Princess Margaret Cancer Centre, Department of Medical Oncology and Hematology, University of Toronto, Toronto, ON M5G 2C1, Canada; Marcus.Butler@uhn.ca; 6 Odette Cancer Centre, Sunnybrook Health Sciences Centre, University of Toronto, Toronto, ON M4N 3M5, Canada; teresa.petrella@sunnybrook.ca; 7 Division of General Surgery, University of Ottawa, Ottawa, ON K1N 6N5, Canada; cnessim@toh.ca; 8 Division of Dermatology, University of Alberta, Edmonton, AB T6G 2R3, Canada; tsalopek@ualberta.ca (T.G.S.); r.gniadecki@ualberta.ca (R.G.)

**Keywords:** targeted therapy, reflex testing, *BRAF* inhibitor, *BRAF* mutation, MAPK pathway, metastatic melanoma, advanced melanoma, stage II

## Abstract

**Simple Summary:**

Reflex molecular testing is an emerging concept in oncology that, for a variety of cancers, was demonstrated to reduce the time to treatment initiation, thus potentially impacting survival outcomes. In advanced melanoma, *BRAF* mutation testing is critical in predicting treatment response with targeted therapy (i.e., BRAF/MEK inhibitors). Certain features were identified in melanomas that harbor *BRAF* mutations (e.g., primary lesions located on the trunk, diagnosed in patients <50, visibly pigmented tumors and, at times, with ulceration or specific dermatoscopic features). For select advanced melanoma patients, delays in determining mutational status present a significant barrier to the prompt initiation of treatment, which can adversely impact patient outcomes, especially in the metastatic setting due to a rapidly progressive disease. Treatment in these cases needs to start promptly by a medical oncologist. Ordering *BRAF* testing by preceding members of the treating team will allow medical oncologists to initiate treatment at the first visit. According to poor survival outcomes, we propose that patients with thick tumors (>4.0 mm) or >2 mm tumors with ulceration (i.e., stage ≥IIB) should potentially be considered for systemic therapy, thus justifying reflex *BRAF* testing. We overview current *BRAF* mutation testing recommendations and methods used in the United States, Canada, and Europe.

**Abstract:**

Targeted therapy has been developed through an in-depth understanding of molecular pathways involved in the pathogenesis of melanoma. Approximately ~50% of patients with melanoma have tumors that harbor a mutation of the *BRAF* oncogene. Certain clinical features have been identified in *BRAF*-mutated melanomas (primary lesions located on the trunk, diagnosed in patients <50, visibly pigmented tumors and, at times, with ulceration or specific dermatoscopic features). While *BRAF* mutation testing is recommended for stage III–IV melanoma, guidelines differ in recommending mutation testing in stage II melanoma patients. To fully benefit from these treatment options and avoid delays in therapy initiation, advanced melanoma patients harboring a *BRAF* mutation must be identified accurately and quickly. To achieve this, clear definition and implementation of *BRAF* reflex testing criteria/methods in melanoma should be established so that patients with advanced melanoma can arrive to their first medical oncology appointment with a known biomarker status. Reflex testing has proven effective for a variety of cancers in selecting therapies and driving other medical decisions. We overview the pathophysiology, clinical presentation of *BRAF*-mutated melanoma, current guidelines, and present recommendations on *BRAF* mutation testing. We propose that reflex *BRAF* testing should be performed for every melanoma patient with stages ≥IIB.

## 1. Introduction

Melanoma incidence and mortality are continuously increasing in the United States, Canada, and other countries around the world [1,2,3,4]. Advances in our understanding of molecular pathways have led to improvements in the historically unfavorable prognosis of metastatic melanoma [5]. One of the most studied regulatory signaling pathways is the mitogen-activated protein (MAP) kinase pathway. In the early 2000s, it was discovered that many cases of metastatic melanoma exhibited inappropriate activation of this pathway through a mutated *BRAF* oncogene [6]. Since then, the development of targeted therapies to suppress this signaling have given *BRAF*-mutation status a critical role in the clinical decision making for the treatment of advanced melanoma. 

Despite the importance of the MAP kinase pathway in the treatment of melanoma, there is no consensus at which time point *BRAF* mutation testing should take place during the workup of melanoma. For some patients, delays in determining mutational status present a significant barrier to the prompt initiation of treatment, which can adversely impact patient outcomes, especially in the metastatic setting, where patients may have a rapidly progressive disease. Treatment in these cases needs to start promptly at the time of diagnosis. This positional paper provides an overview of the pathophysiology and clinical presentation of *BRAF*-mutated melanoma and presents current guidelines and recommendations for *BRAF* mutation testing.

## 2. Pathophysiology

Compared to other types of cancer, melanoma tumor cells have one of the highest frequencies of mutational burden [7], which results from extensive exposure to carcinogenic ultraviolet radiation [7]. Extensive studies summarizing the occurrence of pathogenic mutations in melanoma have been reviewed elsewhere [8,9,10]. Such mutations are commonly found in the MAP kinase signaling pathway, which regulates cellular processes including cell growth, proliferation, and survival [11].

## 3. MAP Kinase Pathway

**Figure 1:**
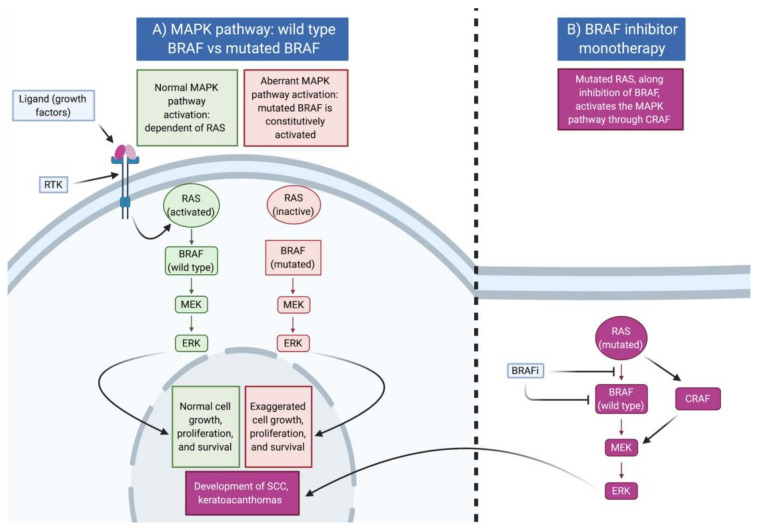
Overview of the MAPK pathway. (**A**) MAP kinase pathway in the settings of wildtype *BRAF* and mutated *BRAF*. In the presence of a *BRAF* mutation, inappropriate constitutive activation of the pathway occurs, leading to cell proliferation. (**B**) In the presence of *BRAF* inhibitor monotherapy, paradoxical activation of the MAP kinase pathway occurs through mutated *RAS* and *CRAF*, an isoform of *BRAF*. *SCC*: squamous cell carcinoma; *RTK:* receptor tyrosine kinase.

The MAP kinase pathway is a signal transduction pathway that transfers an extracellular signal to the nucleus of the cell in order to regulate gene expression [11] (Figure 1). The initial step in the pathway is the binding of a ligand, a growth factor, to the extracellular portion of its cognate receptor tyrosine kinase (RTK) [11]. This leads to activation of the downstream signaling cascade composed of the G-protein RAS, followed by the protein kinases BRAF, MEK, and ERK [11]. Finally, activated ERK migrates to the nucleus and activates various transcription factors involved in the growth, proliferation, and survival of the cell [11]. Mutations leading to constitutive activation of this pathway lead to the inappropriate proliferation of melanocytes [12]. In conjunction with other dysregulated pathways, proliferating melanocytes may then progress to melanoma [12].

Amongst activating mutations of the MAP kinase pathway, up to 50% of cases involve the *BRAF* gene [13,14]. In a majority of cases, *BRAF* mutation involves a substitution of valine (V) at the gene’s 600th codon [15]. By far, glutamic acid (E) is the most frequently substituted amino acid, with an incidence of up to 90% [15]. In vitro, *BRAF*^V600E^ demonstrates a 500-fold increase in kinase activity, which allows for aberrant cell proliferation [16]. Less frequently (<9%), *BRAF* mutations may involve substitutions of the same codon with other amino acids (V600K, V600R, V600M, and V600D) or include substitutions at other positions of the *BRAF* gene (L597V, K601E, and D594N), but these are rare events (<1%) [15,17,18].

To target aberrant MAP kinase pathway signaling, *BRAF* inhibitors (BRAFi) such as vemurafenib, dabrafenib, and encorafenib were developed. Regrettably, BRAFi monotherapy resulted in the development of tumor resistance and relapse in approximately half of the patients within ~6 months [17,18,19]. This occurs through a multitude of *BRAF*-independent mechanisms that allow melanoma cells to maintain MAP kinase signaling [20]. Thus, an attempt to mitigate the limitations and potential harmful consequences of isolated BRAF inhibition was made through the addition of a concomitant MEK inhibitor. A combination therapy consisting of a BRAF and an MEK inhibitor has proven to be advantageous [21,22]. 

Furthermore, BRAFi monotherapy often led to the development of secondary cancers such as squamous cell carcinomas and keratoacanthomas in up to 20% of patients [23]. This phenomenon was found to be the result of paradoxical activation of the MAP kinase pathway in pre-existing keratinocyte lesions with wildtype *BRAF*, but with an activating mutation of *RAS* [24]. The paradoxical activation occurs through cRAF, an isoform of BRAF that is able to independently activate the downstream cascade of MAP kinases (MEK and ERK) [25]. Further studies showed that monotherapy with BRAFi accelerated the development of other pre-existing *RAS* mutation malignancies such as leukemia and pancreatic and colon cancers [26]. The addition of an MEK inhibitor blocks this pathway and decreases the toxicity from RAS activation in normal cells.

Notably, according to previous investigations, the co-occurrence of mutations in *NRAS* and *BRAF* genes has been reported [27]. These mutations are not mutually exclusive. However, as per the National Comprehensive Cancer Network (NCCN) guidelines (version 2.2021), there is a low probability that they would occur together [28]. The concomitant mutation of *BRAF* and *NRAS* genes may lead to a resistance to MEK inhibition [27]. However, the current evidence is insufficient to suggest that the presence of a co-mutation would require a change in recommended therapy [28].

## 4. Clinical Presentation of *BRAF*-Mutated Melanomas and the Use of *BRAF* as a Prognostic or Treatment Response Predictive Marker

Several clinical features have been identified in melanomas harboring a mutation in *BRAF* (Table 1). It is important to note that the NCCN guidelines do not recommend the use of these features for the determination of mutation status or to make decisions regarding testing [29]. Typically, younger patient age was found to be associated with the presence of a *BRAF* mutation [6,30,31], as well as high estimated annual life UV exposure [28,32], higher total body nevus counts [28], fewer markers of chronic sun damage (i.e., absence of solar elastosis) in the surrounding skin [6,30], and the presence of ulceration [14]. Furthermore, on dermoscopy, findings of irregular peripheral streaks [31], blue-white veil [33], and “peppering” (the latter representing regression and melanophages in the dermis) [34] were associated with *BRAF* mutation in patients. Hence, primary lesions that are located on the trunk, diagnosed before the age of 50, visibly pigmented and, at times, with an ulceration have been shown to have an association with mutated *BRAF* [6,32]. Of the subtypes of cutaneous melanoma, mutated *BRAF* is seen most frequently in superficial spreading and nodular subtypes [6,30,35]. Some reports have also found *BRAF* mutation status to be associated with an increased tumor thickness [36].

Notably, *BRAF* mutation status is currently the only validated predictive treatment response marker in melanoma [37,38,39]. The presence of a mutation is highly predictive of response to therapy with BRAF/MEK inhibition [37]. However, the presence of the *BRAF* mutation is not a useful prognostic marker for melanoma. The disease-free interval from primary diagnosis to first metastasis appears to be no different based on *BRAF* mutation status alone [6]. Overall survival for patients with primary melanoma harboring *BRAF* mutations also does not appear to be impacted [6,36]. Studies examining the outcomes of survival in metastatic disease have yielded conflicting results [36]. Some studies reporting reduced overall survival in *BRAF*-mutated melanoma were confounded by factors such as the tendency of *BRAF*-mutated melanoma to present at more advanced disease stages [30,35] and the eligibility of patients to received targeted therapy in clinical trials [6]. Although there is the perceived notion that *BRAF*-mutated melanoma has a more aggressive clinical course, this has not been established in clinical studies [36,40]. 

## 5. *BRAF* Testing at the Time of Diagnosis

### 5.1. Overview of Diagnosis

The definitive diagnosis of melanoma requires histopathologic assessment of the tumor. Based on the eighth edition of the American Joint Committee on Cancer (AJCC) staging system, parameters of the primary tumor (T), lymph nodes and lymphatic drainage (N), and distant metastases (M) are used to determine the pathologic stage (Table S1). Patients with primary tumors without spread are classified as stage I or II, depending on the tumor characteristics (tumor thickness and ulceration only). Tumors that have spread beyond the primary skin site as indicated by the presence of in-transit tumors, satellite tumors, or involvement of lymph nodes, but without distant metastases are classified as stage III. Patients with distant metastases are categorized as stage IV. Each stage carries a different risk of disease relapse and survival [41].

**Table 1:** Frequently reported features of melanoma found to be associated with *BRAF* mutation status.

Patient Features	Primary Melanoma	Metastatic Melanoma
BRAF mutation prevalence	Primary melanoma: 33–47% [6]	Metastatic melanoma: 41–55% [6]
	Recurrent melanoma found to have higher frequency of *BRAF* mutation [14]	-
**Patient Features**		
Age of diagnosis	<50 [6,30]	Younger individuals [6]
UV exposure	High estimated lifetime exposure [28] and early-life exposure [28,32]	-
Total body nevus count	Patients with high number of nevi on back (>14) [28] more likely to harbor a *BRAF* mutation	-
Chronic sun-damaged skin	Fewer signs of chronic sun damage [30], such as lentigines [32] and solar elastosis [14,28]	Less chronic sun damage [6]
**Melanoma Features**		
Number of primary lesions	-	Occult or 1 lesion [6]
Location of primary melanoma	Truncal location [6,30,41]	Truncal location [6]
Melanoma subtype	Superficial spreading [30] or nodular [14]	-
Pigmentation	Presence of pigmentation on pathology and as detected by patient [32]	-
Breslow thickness (of primary)	*BRAF* mutation associated with increased tumor thickness compared to wildtype [42,43]	-
Ulceration (of primary)	*BRAF* mutation associated with the presence of ulceration [14,41,44,45]	No association [6]
Dermoscopy features	Irregular peripheral streaks [31], blue-white veil [33], and “peppering” [34]	*-*
**Outcomes**		
Stage at presentation	Presentation at a more advanced stage is associated with *BRAF* mutation [30,35]	
Response to chemotherapy	-	No association [6]
Response to BRAF/MEK inhibitor	-	Highly predictive of response to therapy [37]
Disease-free interval (primary diagnosis to first distant metastasis)	-	No association [6]
Outcome (survival)	No association [6]	Further investigation necessary

Importantly, variability exists in the published guidelines directing *BRAF* mutation testing. The NCCN guidelines recommend *BRAF* testing in patients for whom targeted therapy may be an option [29]. This includes patients with stage III melanoma at high risk for recurrence or patients presenting with loco-regional recurrence or stage IV disease. The NCCN panel does not recommend *BRAF* testing for resected pathologic stage I or II cutaneous melanoma unless the results may be used to direct participation in clinical trials. The European Society for Medical Oncology (ESMO) mandates mutation testing for all patients with advanced disease, which includes stages III or IV (resected or unresected) [46]. Contrary to the NCCN guidelines, ESMO recommends mutation testing for high-risk pathologic stage IIC melanoma patients.

As the landscape for treatment options expands, clear guidelines for biomarker testing ensure that high-risk patients receive the first-line treatment options for which they are eligible. As mentioned, there is currently a discrepancy between the published guidelines. In congruence with the ESMO guideline recommendation for testing, pathologic stage IIC should be recognized as high-risk melanoma, and these tumors should undergo mutation testing. This is supported by the evidence/clinical data reporting that stage IIC melanoma patients have paradoxically worse outcomes of overall survival (OS) and relapse-free survival (RFS), when compared to patients presenting at stage IIIA [47,48]. Specifically, 5-year survival rates for both stage IIB and stage IIC disease (87% and 82%, respectively) are lower than the 5-year survival rate of 93% for stage IIIA melanoma [41,47]. Although targeted or immunotherapies are not currently formally indicated in high-risk stage II patients, a number of ongoing clinical trials (e.g., MK-3475-716/KEYNOTE-716 and CheckMate76K trials) will aid to resolve the role of adjuvant therapy in pathologic stage IIB/C disease. Hence, patients with thick tumors (>4.0 mm) or >2 mm tumors with ulceration should potentially be considered for systemic therapy, thus justifying reflex *BRAF* testing in this higher-risk patient population.

### 5.2. Methods of BRAF Mutation Testing

Many testing options are available to detect BRAF mutations, each with unique strengths and weaknesses to be taken into consideration. The current guidelines do not provide a detailed diagnostic testing algorithm. In clinical practice, in some centers, immunohistochemistry (IHC) can be used as a preliminary screening tool to initiate treatment. Confirmatory testing can then be performed using molecular techniques, while other centers prefer the use of real-time PCR (RT-PCR) or next-generation sequencing (NGS) approaches to detect mutation over IHC. Notably, while, in Canada, confirmatory/validation testing can be performed within a hospital testing center using a locally accepted technology, in the United States, only specific platforms are certified by the Food and Drug Administration (FDA) to confirm BRAF mutation status. In Europe, according to the ESMO guidelines, a validated test should be used only in an accredited (certified) institute that includes appropriate quality controls [49]. A summary of the diagnostic testing modalities is provided in Table 2.

### 5.3. Immunohistochemistry (IHC)

Binding of the monoclonal antibody VE1 allows for the detection of the mutant BRAF V600E protein. This approach is preferred in many European centers and is able to effectively and efficiently identify the presence of the mutated protein in formalin-fixed and paraffin-embedded (FFPE) tissue samples. The antibody is a qualitative tool that has not been validated for quantifying the level of expression of the mutated protein [50]. It is a relatively low-cost test with fast turnaround time for results and a reported sensitivity as high as 98.6% and a specificity of 97.7% [51]. Thus, IHC may serve as a cost-effective first-line screening method for BRAF mutations providing sufficient evidence to allow patients to begin targeted therapy [29]. However, even if results of IHC are positive and therapy begins, the NCCN recommends confirmatory molecular testing to be performed [29]. Given that the antibodies used in IHC currently are limited to only the V600E mutation, negative IHC results require further testing with molecular-based modalities to assess for other BRAF variants/mutations that can benefit from targeted therapy. 

### 5.4. Real-Time PCR

RT-PCR is used to amplify tumor DNA sequences and tags mutant and wildtype sequences with labeled primers. Comparing the strength of the wildtype and mutant signal determines the presence of mutation [50]. This technique is relatively fast and cost-effective and has demonstrated sensitivity and specificity as high as 96% and 100%, respectively [50]. However, this technique is often used with kits that contain primers that were only designated to target the most common mutations including V600E, K, and D. Although, the US FDA approved Cobas^®^ 4800 and THxID^®^ assays to be used as a confirmatory test for *BRAF* mutation, specialized treatment centers generally tend to use genomic sequencing to be able to also detect mutations that involve other loci and genes [50].

**Table 2:** Summary of diagnostic testing modalities used to detect *BRAF*-mutated melanoma. IHC, immunohistochemistry; HRM, high-resolution melt; NGS, next-generation sequencing; RT-PCR, real-time polymerized chain reaction.

Features	IHC	RT-PCR		HRM	Sanger	Pyrosequencing	NGS
		Cobas^®^	THxID^®^				
Detection of mutations [50,52]	VE1 antibody for V600E	V600E	V600E V600K	Indirectly detects mutations	Whole exon, detects rare mutations	Optimized for V600 mutations	Whole exon, detects rare mutations
Sensitivity	Up to 98.6% [51]	95% [53]	>96% (V600E) >92% (V600K) [50]	99% [52]	92.5% (for V600E) [53]	90 to 100% [52,54]	99% [55]
Specificity	97.7% [51]	98% [50]	100% [50]	100% [52]	100% [52]	95 to 100% [52,54]	100% [55]
Limit of detection (i.e., proportion of cells that are positive)	Few cells [56]	7% [52]	5% [50]	6.6% [50]	6.6% [57]	5.0% [58]	2% [52]
Turnaround time [52]	<1 day	1 day		1 day	Up to 3 days	2 days	Up to 5 days
Cost [52]	Low	Medium		Low	Medium	High	Very high

It is worth noting that a recently developed fully automated *BRAF* mutation RT-PCR test (IdyllaT^M^ by Biocartis) is able to detect BRAF V600E, E2, D, K, R, and M mutations with high sensitivity. This test identifies mutated cells representing only 1% in the wildtype background (detection limit 1%) in FFPE samples [59]. This method also has the benefit of having a fast turnaround time (~2 h) as no DNA extraction step is required since 5–10 μm FFPE sections are loaded directly into the device. The disadvantages include higher cost of the equipment and the limitation that only one sample can be processed at a time.

### 5.5. High-Resolution Melt

High-resolution melt curve analysis (HRM) is a PCR-based method that uses the melting temperature of PCR products to determine mutations in DNA sequences. In general, this results in rapid turnaround time at a low cost [50]. Although some studies reported variable sensitivity and specificity for this technique, a meta-analysis of melanoma and other *BRAF*-mutated cancers demonstrated that the pooled sensitivity for HRM in melanoma was ~99% (ranging from 93% to 100%) and pooled specificity was ~99% (ranging from 88% to 100%) [60]. Similar to RT-PCR, the disadvantage of HRM is that the direct identification of the specific nucleotide sequence is not possible. Several centers use IHC and, subsequently, HRM and RT-PCR molecular methods to confirm *BRAF* mutation findings. If the molecular results are equivocal, Sanger sequencing or next-generation sequencing is then employed to establish the mutation status.

### 5.6. Sanger Sequencing

Sanger sequencing, less commonly used today, was historically regarded as the gold standard for the identification of acquired mutations. It determines a complementary sequence of DNA after various lengths of the sequence are produced with labeled nucleotides [50]. This allows for the identification of other mutation sequences of *BRAF* and, thus, is not only limited to the V600E mutations, as observed in IHC and RT-PCR tests. However, this technique requires a high percentage of tumor cells within a sample. This may necessitate pathologists to perform a macrodissection of a sample if the tumor cell percentage is <50% [61]. The sensitivity of Sanger sequencing is reported at 92.5% for the V600E mutation [53], and it has a specificity of up to 100% [61]. As it offers a relatively low sensitivity (with a high limit of detection ~20% [61]) at a mid-range cost and turnaround time, Sanger sequencing is generally not considered to be a reference test for *BRAF* mutation status [61], but can rather be used as a confirmatory test (i.e., if results of PCR-based testing are negative or inconclusive), although it is not commonly used in current practice [50,62].

### 5.7. Pyrosequencing

The process of DNA synthesis is also utilized in pyrosequencing; however, by detecting enzymatic reactions with each addition of a base pair, this process is generally faster compared to Sanger sequencing. Pyrosequencing has also been found to have a lower limit of detection than Sanger sequencing and a sensitivity of 98%. However, it has been reported to have a lower specificity, ranging from 90% to 100% [50]. This relatively newer technology can be expensive, as costs of equipment and reagents are considerably high. Although the overall processing time is longer than IHC and RT-PCR, it is able to perform more detailed genomic sequencing faster than the Sanger sequencing method.

### 5.8. Next-Generation Sequencing (NGS)

The use of multigene analysis is expected to become more prevalent in melanoma management due to its high mutational burden. Over the past decade, NGS has increasingly became the choice of large-scale sequencing. NGS can detect additional gene variants, quantify variant allele frequency, and analyze multiple genes with great sensitivity (99%, limit of detection 2%) and specificity (100%) [55]. Importantly, it is possible to analyze samples with limited tumor tissue present, unlike traditional Sanger sequencing [61]. However, the impressive features of NGS come at a high cost and require more hands-on processing time than other methods [52]. Limiting the analysis of NGS to actionable genes such as *BRAF* was shown to be cost- and time-effective [61]. From a research perspective, collecting information on other mutation drivers is of interest to identify potential future therapeutic targets and to select patients for clinical trials.

## 6. Implementation of Reflex Testing

Despite major advances in the laboratory turnaround time for the aforementioned diagnostic modalities, there remain significant barriers to the timely implementation of personalized medicine. In the workup of advanced melanoma, patients often require referral/input from a diverse range of specialists, which may include dermatologists, surgeons, pathologists, and medical oncologists. The process of selecting which melanomas require further testing is the step that produces a significant delay, especially if tissues require relocation to a different testing center. If advanced melanoma patients arrive to the medical oncologist appointment without *BRAF* mutation status, this ultimately translates to unnecessary wait times before the treatment can be initiated. For advanced melanoma, ordering *BRAF* testing by preceding members of the treating team will allow medical oncologists to initiate therapy promptly, which may impact disease outcomes.

Reflex molecular testing is an emerging concept in medical oncology. In particular, it is valuable for biomarkers that are not universally ordered for all presentations of cancer (Table 3). In breast cancer, all tumors are sent for hormone receptor and human epidermal growth factor (*HER2*) receptor testing, regardless of whether they are primary, recurrent, or metastatic. However, not all melanoma biopsies are tested for *BRAF* mutation, as primary cutaneous melanomas are rarely managed with systemic therapies. Thus, the development of clear *BRAF* reflex testing criteria/guidelines can prevent unnecessary waiting periods.

In non-small-cell lung cancer (NSCLC), the use of reflex testing has been demonstrated to successfully reduce the time to treatment initiation. Cheema et al. [63] implemented a model of reflex testing in NSCLC, where pathologists reflexively order biomarker tests (*EGFR* and *ALK*) immediately upon pathological confirmation of NSCLC diagnosis. Patients were then able to more consistently arrive to their first medical oncology appointment with known biomarker status. Effectively, this reduced the median time to treatment initiation by 21 days [63].

Similar models of reflex testing in lung, breast, colon, and ovarian cancers have also successfully demonstrated a reduction in time to treatment initiation. While further discussion on reflex testing in other cancers is beyond the scope of this paper, we present a summary of how reflex testing can be used to make therapeutic (Table 3) or other medical decisions (Table 4).

Lastly, the results of reflex testing on survival have yet to be fully elucidated. Metastatic melanoma disseminates quickly and has a grim prognosis if left untreated; patients with stage IV melanoma have a reported median survival of only 7.5 months [64]. Reduction in the delay of treatment initiation may lead to a favorable survival outcome in advanced melanoma patients. Following implementation of reflex *BRAF* testing in melanoma, it would be of interest to study whether there is indeed an effect on time to treatment initiation and/or impact on survival outcomes.

## 7. Treatment

As per the NCCN, the best management of any patient with advanced cancer is through participation in a clinical trial (category 2A recommendation) [29], although, in recent years, a number of therapies have been vigorously vetted and proven effective for advanced melanoma. Current trials are investigating the role of combining immunotherapy and targeted therapy and determining optimal sequencing of treatments if used consecutively [22,65]. Investigations of the utility of early adjuvant therapy in early stages of melanoma are also underway, in an effort to potentially eradicate residual disease before it becomes overtly metastatic. Early adjuvant therapy use would be ideally combined with a stronger comprehension of biomarkers predicting tumor aggressiveness [66].

For advanced metastatic melanomas, immune-checkpoint inhibitors and targeted therapies are now approved as first-line treatments [29]. There is currently a lack of clinical trials directly comparing immune-checkpoint inhibitors, targeted therapy, or a combination of both for patients with *BRAF* mutated melanomas. Thus, there are no clear directives for which treatment should be used first-line for patients, who may be eligible for both. Decisions directing treatment should always be informed on a case-by-case basis. The current recommendations based on the NCCN guidelines are presented in Table 5.

Notably, for patients with documented *BRAF* V600 mutation, targeted therapy becomes an important first- or second-line systemic option. Due to the paradoxical development of resistance, relapse, and secondary cancers in BRAFi monotherapy [17,18,19], targeted therapy treatments now include the addition of an MEK inhibitor. Combination with MEK inhibition reduces rates of resistance and provides long-term survival benefit of up to 5 years, especially in patients with normal lactate dehydrogenase (LDH) levels and fewer than three sites of disease [17,18,19,21,67,68,69]. Multiple phase III studies have confirmed the superior benefit of *BRAF/MEK* inhibitor combination therapy for PFS and OS in patients with unresected or metastatic melanoma (Table S2)**.** Combination of *BRAF/MEK* inhibitors has also demonstrated improved PFS and OS, when used as adjuvant therapy for resected stage III melanoma [17,18,19,21,67,68,69].

On the basis of these favorable results, the NCCN (version 2.2021) recommends combined targeted therapy (i.e., dabrafenib/trametinib) as adjuvant treatment for all patients with stage III disease harboring an activating mutation of *BRAF* V600 [29]. However, due to limited trial evidence of efficacy in resected stage IV disease, adjuvant *BRAF/MEK* inhibitor therapy is not currently recommended for these patients. For unresected or distant metastatic disease harboring *BRAF* V600 mutation, first-line options for *BRAF/MEK* inhibition include dabrafenib/trametinib, vemurafenib/cobimetinib, or encorafenib/binimetinib [29].

**Table 3:** Roles of predictive biomarkers in various types of cancer. Those presented below have studies examining the effect of reflex testing on direct management options (primarily targeted therapy). Biomarker and predictive values were adapted from El-Deiry et al. [70]; other sources used are cited directly in text. DDR, DNA damage response; EGFR, epidermal growth factor receptor; ER, estrogen receptor; HER2, human epidermal growth factor receptor 2; NSCLC, non-small-cell lung cancer; PARP, poly (ADP-ribose) polymerase; PR, progesterone receptor.

Malignancy	Biomarker	Predictive Value	Patient Population	Reflex Testing Used	Outcomes Observed
Breast cancer	Oncotype Dx multigene assay	Predictive of chemotherapy benefit	Stage I, II *ER*+/*PR*+/*HER2*−	Reflex testing criteria developed for surgeons to order the test immediately after post-operative pathology results are available [71]	Incorporation of Oncotype DX testing reduces unwarranted chemotherapy use, improves life expectancy, and is cost-effective [72]. The introduction of reflex criteria testing for surgeons to implement reduced time from surgery to initiation of chemotherapy by 6.4 days [73]
Colon cancer	*KRAS*	Predictive for resistance to anti-EGFR therapy	Patients evaluated for metastatic disease, whenever anti-EGFR therapy is considered	Reflex *KRAS* testing is requested in metastatic cases of colon cancer starting second-line therapy [74]	Reflex testing offers maximal lead time to identify patients suitable for third-line anti-EGFR therapy [74]
Lung cancer (NSCLC)	*EGFR ALK*	Positive predictor of treatment with EGFR tyrosine kinase inhibitors or ALK tyrosine kinase inhibitors, respectively	Patients with advanced lung cancer who are candidates for targeted therapy. The NCCN recommends molecular profiling for all patients with metastatic NSCLC.	Reflex testing of ALK and EGFR by pathologists at the time of diagnosis of NSCLC [63]	Reduces the median time to treatment using systemic therapy by 10 days [63]
	*EGFR*, *ALK*, *ROS1*, *MET*, *RET*, *KRAS BRAF*, *PDL1*, *HER2*	*EGFR* and *ALK* have the greatest evidence supporting targeted therapy for mutations. Rearrangements in other genes have lower-level evidence to direct management	Patients with newly diagnosed lung adenocarcinoma of any pathologic stage	Retrospective examination of the effect of reflex testing of molecular biomarkers at the time of pathologic diagnosis of lung adenocarcinoma [44]	Reduces the average turnaround time of testing by 26 days and almost doubles the rate of variants that are detected [44]
Ovarian cancer	*BRCA1* *BRCA 2*	Predictive of response to PARP inhibitor and eligibility for genetic counseling	Women with high-grade serous carcinoma are eligible for *BRCA* mutation testing	Reflex tumor testing of all high-grade serous carcinoma at initial diagnosis [45]	Reflex testing identifies more *BRCA* mutations, reduces the time to critical treatment decision, and helps to determine other *BRCA* mutation carriers that may benefit from preventative treatment [45]
Prostate cancer (castration resistant)	*BRCA 1* *BRCA 2* *ATM*	Predictive of response with PARP and other DDR enzyme inhibitors	Men with metastatic prostate cancer	Suggestion to examine whether men with earlier-stage disease may benefit from reflex testing strategies [75]	Yet to be tested

**Table 4:** Roles of predictive biomarkers in various types of cancer. Those presented below are examples of the utility of reflex testing for purposes primarily outside of direct clinical management (e.g., genetic counseling). HPV, human papillomavirus; IHC, immunohistochemistry; MSM, men who have sex with men; NCCN, National Comprehensive Cancer Network.

Malignancy	Biomarker	Purpose	When to Test	Utility of Reflex Testing
Anal squamous cell carcinoma	HPV	Screening test for anal squamous cell carcinoma (SCC)	Annual rectal exam in high-risk groups such as MSM	Reflex testing of HPV for high-risk patients (HIV+ and other immunocompromised individuals) to screen for anal squamous cell carcinoma
Chronic myeloid leukemia (CML)	*BCR*-*ABL*	Establish initial patient baseline level and assess response to therapy in follow-up samples	As part of workup for CML or acute lymphoblastic leukemia (ALL)	Following a positive BCR-ABL1 RT-PCR result, a reflex test is performed to provide a quantitative measurement of BCR/ABL1 mRNA transcript to be recorded as the baseline level [76]
Chronic myeloid leukemia (CML) Acute myeloid leukemia with myelodysplasia-related changes (AML-MRC)	*KIT*	Identifying the co-occurrence of systemic mastocytosis	Patients diagnosed with CML or AML-MRC, with an identified D816V mutation of *KIT*	Identifying systemic mastocytosis with associated hematologic malignancy allows for appropriate treatment of the systemic mastocytosis component [77]
Colon cancer	Mismatch repair genes	Genetic counseling to identify patients with Lynch syndrome and also predictive of response to immune-checkpoint inhibitors	As detailed in the Bethesda testing guidelines for Lynch syndrome	Ontario is performing reflex IHC in colorectal cases presenting before the age of 40 [78] to identify Lynch syndrome patients
Endometrial cancer	Mismatch repair genes	Detection of Lynch syndrome	As detailed in the Bethesda testing guidelines for Lynch syndrome	Implementation of reflex testing of all newly diagnosed endometrial cancers with IHC is suggested to identify patients, who are at high risk and could benefit from prevention strategies [79]
Head and neck squamous cell carcinoma	HPV	Positive prognostic and predictive marker of response to treatment	Patients with newly diagnosed oropharyngeal squamous cell carcinoma	Reflex testing of oropharyngeal primary tumors with p16 IHC [80]
Pancreatic cancer	*BRCA1* *BRCA2*	Genetic counseling to identify other potential carriers of founder mutations. Predictive of response to PARP inhibitors	All patients with pancreatic cancer (NCCN guidelines)	Reflex testing of founder mutations recommended for patients with pancreatic adenocarcinoma with French Canadian or Ashkenazi Jewish ancestry [81]

There is currently weak evidence suggesting that targeted therapy has better immediate outcomes, whereas immune-checkpoint inhibitors may have a more durable long-term response [46]. Despite durability, the response to immune-checkpoint inhibitors may be slower than with targeted therapy [29]. The NCCN guidelines suggest that *BRAF*/*MEK* inhibition may be preferred for patients who may benefit from a more rapid response [29]. Of course, to achieve this, it would be ideal to obtain *BRAF* testing results prior to the first appointment with a medical oncologist. This is especially important in patients who have a personal history of significant autoimmune disease (e.g., systemic lupus erythematosus, psoriasis, or inflammatory bowel disease) or other comorbidities (e.g., solid organ transplant recipients) that may preclude the use of immunotherapy. As *BRAF*-mutated tumors tend to present at more advanced stages, early treatment with appropriate agents is crucial to allow for potential tumor regression and improvements in the quality of life [6,82]. While the NCCN guidelines indicate that both immunotherapy and targeted therapies are appropriate first-line treatments in advanced disease, ESMO specifically suggests that immunotherapy should be considered first-line over targeted therapy in *BRAF*-mutated melanoma in the absence of rapidly progressing tumors or tumors threatening important organs and/or function. Targeted therapy can then be reserved for subsequent lines of treatment thereafter [46].

## 8. Recommendation on *BRAF* Reflex Testing

The implementation of carefully developed disease-specific reflex testing criteria by a multidisciplinary team is important to avoid the futile use of valuable healthcare resources. For *BRAF* mutation in the context of melanoma, reflex testing criteria should include advanced disease characteristics, as these patients would benefit the most from rapid initiation of *BRAF/MEK* inhibitors. These features to a clinician/pathologist might include melanomas exhibiting clinical characteristics associated with *BRAF* mutation (summarized in Table 1), thick tumors of Breslow depth 2–4 or >4 mm with or without ulceration (i.e., stages IIB and IIC, respectively) and all patients with nodal involvement (i.e., stage III) or lymphatic progression (satellitosis or in transit metastasis). While systemic therapies are not approved for patients with pathologic stage II melanoma, considering the risk of disease progression in these individuals and decreased 5- and 10-year survival rates (82% and 75%, respectively, for stage IIC and 87% and 82%, respectively, for stage IIB disease), knowledge of the *BRAF* mutational status may prove useful for selection of future therapies. Furthermore, if stage IIB/C melanoma recurs, this usually occurs within 2 years of surgery. Advanced knowledge of the mutation status will help initiate treatment faster for newly metastatic or recurrent disease. While many tertiary care centers and specialized melanoma programs have or are actively implementing reflex *BRAF* mutation testing, it is paramount to promote this change across community hospitals as well, so that patients with high-risk (stage ≥IIB) melanoma can consistently arrive to their first medical oncology appointment with this information at hand to make an informed treatment decision. This may be critically important for those patients who present to the multidisciplinary clinic with far more advanced melanomas than implied by the microstaging features of the primary tumor. For example, patients with large infiltrating tumors of dubious resectability or tumors that involve vital structures might benefit from neoadjuvant targeted therapy to facilitate their removal. Furthermore, as noted earlier, ongoing clinical trials (MK-3475-716/KEYNOTE-716, and CheckMate76K that enrolled stage IIB and IIC patients) should answer the question of whether these patients might benefit from adjuvant targeted therapy. The collective agreement on worrisome signs identifiable by dermatologists, surgeons, pathologists, and oncologists will enable cost-effective reflex *BRAF* testing and timely management for patients.

**Table 5:** Summary of treatment recommendations adapted from the NCCN guidelines. Strength of recommendations are between 1 and 2A. (NCCN Recommendation Categories: 1—based upon high level-evidence, there is uniform NCCN consensus that the intervention is appropriate; 2A—based upon lower-level evidence, there is uniform NCCN consensus that the intervention is appropriate; 2B—based upon lower-level evidence, there is NCCN consensus that the intervention is appropriate; 3—based upon any level of evidence, there is major disagreement that the intervention is appropriate).

Stage	*BRAF* Status	Tumor	Recommended Systemic Treatment Options
I	Any	Resected	None
II	Any	Resected	None
III	Wildtype (adjuvant)	Resected	Nivolumab Pembrolizumab Ipilimumab ^#,^*
	Wildtype (therapeutic)	Unresected	Nivolumab Pembrolizumab Ipilimumab * Nivolumab/ipilimumab
	*BRAF*-mutated (adjuvant)	Resected	Nivolumab Pembrolizumab Ipilimumab Dabrafenib/trametinib
	*BRAF*-mutated (therapeutic)	Unresected	Nivolumab Pembrolizumab Ipilimumab * Dabrafenib/trametinib Nivolumab/ipilimumab
IV	Any (adjuvant)	Resected	Nivolumab Pembrolizumab Ipilimumab * Nivolumab/ipilimumab
	*BRAF*-mutated (therapeutic)	Unresected	Nivolumab Pembrolizumab Ipilimumab * Dabrafenib/trametinib Vemurafenib/cobimetinib Encorafenib/binimetinib Nivolumab/ipilimumab

* Ipilimumab is recommended if patient had prior exposure to anti-PD-1 therapy. ^#^ Although the US FDA has approved ipilimumab in the adjuvant setting, in Canada, the manufacturers never sought approval from Health Canada for ipilimumab as an adjuvant treatment.

## 9. Conclusions

Exploitation of the MAP kinase signaling pathway has led to great improvements in the prognosis of metastatic melanoma. Mutational testing of high-risk melanoma gives patients the option of personalized treatment, which has been shown to provide a greater survival benefit than historical treatment modalities. Importantly, the implementation of standardized reflex testing criteria will allow for timely initiation of these treatment options. Further research identifying optimal use of therapies and new molecular targets will continue to improve the outlook for advanced melanoma.

## Supplementary Materials

The following are available online at https://www.mdpi.com/article/10.3390/cancers13092282/s1, Table S1: Simplified overview of the pathologic staging of melanoma (American Joint Committee on Cancer 8th edition), Table S2: Summary of targeted therapy trials in the treatment of unresected stage III or IV melanoma and resected melanoma. *NR*: not reported.

## References

[1] Ghazawi F.M., Cyr J., Darwich R., Le M., Rahme E., Moreau L., Netchiporouk E., Zubarev A., Roshdy O., Glassman S.J. (2019). Cutaneous malignant melanoma incidence and mortality trends in Canada: A comprehensive population-based study. J. Am. Acad. Dermatol..

[2] Ghazawi F.M., Le M., Lagacé F., Cyr J., AlGhazawi N., Zubarev A., Roy S.F., Rahme E., Netchiporouk E., Roshdy O. (2019). Incidence, Mortality, and Spatiotemporal Distribution of Cutaneous Malignant Melanoma Cases Across Canada. J. Cutan. Med. Surg..

[3] Muntyanu A., Savin E., Ghazawi F.M., Alakel A., Zubarev A., Litvinov I.V. (2020). Geographic Variations in Cutaneous Melanoma Distribution in the Russian Federation. Dermatology.

[4] Ghazawi F.M., Le M., Alghazawi N., Rahme E., Moreau L., Netchiporouk E., Zubarev A., Roshdy O., Glassman S.J., Sasseville D. (2019). Trends in incidence of cutaneous malignant melanoma in Canada: 1992-2010 versus 2011-2015. J. Am. Acad. Dermatol..

[5] Petrella T., Ernst S., Spatz A., Claveau J. (2012). Canadian perspective on the clinical management of metastatic melanoma. Lung.

[6] Long G.V., Menzies A.M., Nagrial A.M., Haydu L.E., Hamilton A.L., Mann G.J., Hughes T.M., Thompson J.F., Scolyer R.A., Kefford R.F. (2011). Prognostic and Clinicopathologic Associations of Oncogenic BRAF in Metastatic Melanoma. J. Clin. Oncol..

[7] Lawrence M.S., Stojanov P., Polak P., Kryukov G.V., Cibulskis K., Sivachenko A., Carter S.L., Stewart C., Mermel C.H., Roberts S.A. (2013). Mutational heterogeneity in cancer and the search for new cancer-associated genes. Nat. Cell Biol..

[8] Eddy K., Shah R., Chen S. (2021). Decoding Melanoma Development and Progression: Identification of Therapeutic Vulnerabilities. Front. Oncol..

[9] Ito T., Tanaka Y., Murata M., Kaku-Ito Y., Furue K., Furue M. (2021). BRAF Heterogeneity in Melanoma. Curr. Treat. Options Oncol..

[10] Newton-Bishop J., Bishop D., Harland M. (2020). Melanoma Genomics. Acta Derm. Venereol..

[11] McCain J. (2013). The MAPK (ERK) Pathway: Investigational Combinations for the Treatment Of BRAF-Mutated Metastatic Melanoma. Pharm. Ther..

[12] Shain A.H., Bastian B.C. (2016). From melanocytes to melanomas. Nat. Rev. Cancer.

[13] Burotto M., Chiou V.L., Lee J.-M., Kohn E.C. (2014). The MAPK pathway across different malignancies: A new perspective. Cancer.

[14] Spathis A., Katoulis A.C., Damaskou V., Liakou A.I., Kottaridi C., Leventakou D., Sgouros D., Mamantopoulos A., Rigopoulos D., Karakitsos P. (2019). BRAF mutation status in primary, recurrent, and metastatic malignant melanoma and its relation to histopathological parameters. Dermatol. Pract. Concept..

[15] Bradish J.R., Cheng L. (2014). Molecular pathology of malignant melanoma: Changing the clinical practice paradigm toward a personalized approach. Hum. Pathol..

[16] Wan P.T., Garnett M.J., Roe S.M., Lee S., Niculescu-Duvaz D., Good V.M., Jones C.M., Marshall C.J., Springer C.J., Cancer Genome Project (2004). Mechanical acts RAF-Erk signal pathway by oncological mutations B-Raf. Cell.

[17] Ascierto P.A., McArthur G.A., Dréno B., Atkinson V., Liszkay G., Di Giacomo A.M., Mandalà M., Demidov L., Stroyakovskiy D., Thomas L. (2016). Cobimetinib combined with vemurafenib in advanced BRAFV600-mutant melanoma (coBRIM): Updated efficacy results from a randomised, double-blind, phase 3 trial. Lancet Oncol..

[18] Long G.V., Flaherty K.T., Stroyakovskiy D., Gogas H., Levchenko E., de Braud F., Larkin J., Garbe C., Jouary T., Hauschild A. (2017). Dabrafenib plus trametinib versus dabrafenib monotherapy in patients with metastatic BRAF V600E/K-mutant melanoma: Long-term survival and safety analysis of a phase 3 study. Ann. Oncol..

[19] Robert C., Karaszewska B., Schachter J., Rutkowski P., Mackiewicz A., Stroiakovski D., Lichinitser M., Dummer R., Grange F., Mortier L. (2015). Improved Overall Survival in Melanoma with Combined Dabrafenib and Trametinib. N. Engl. J. Med..

[20] Shi H., Hugo W., Kong X., Hong A., Koya R.C., Moriceau G., Chodon T., Guo R., Johnson D.B., Dahlman K.B. (2014). Acquired Resistance and Clonal Evolution in Melanoma during BRAF Inhibitor Therapy. Cancer Discov..

[21] Robert C., Grob J.J., Stroyakovskiy D., Karaszewska B., Hauschild A., Levchenko E., Sileni V.C., Schachter J., Garbe C., Bondarenko I. (2019). Five-Year Outcomes with Dabrafenib plus Trametinib in Metastatic Melanoma. N. Engl. J. Med..

[22] Gutzmer R., Stroyakovskiy D., Gogas H., Robert C., Lewis K., Protsenko S., Pereira R.P., Eigentler T., Rutkowski P., Demidov L. (2020). Atezolizumab, vemurafenib, and cobimetinib as first-line treatment for unresectable advanced BRAFV600 mutation-positive melanoma (IMspire150): Primary analysis of the randomised, double-blind, placebo-controlled, phase 3 trial. Lancet.

[23] Heidorn S.J., Milagre C., Whittaker S., Nourry A., Niculescu-Duvas I., Dhomen N., Hussain J., Reis-Filho J.S., Springer C.J., Pritchard C. (2010). Kinase-Dead BRAF and Oncogenic RAS Cooperate to Drive Tumor Progression through CRAF. Cell.

[24] Su F., Viros A., Milagre C., Trunzer K., Bollag G., Spleiss O., Reis-Filho J.S., Kong X., Koya R.C., Flaherty K.T. (2012). RASMutations in Cutaneous Squamous-Cell Carcinomas in Patients Treated with BRAF Inhibitors. N. Engl. J. Med..

[25] Montagut C., Sharma S.V., Shioda T., McDermott U., Ulman M., Ulkus L.E., Dias-Santagata D., Stubbs H., Lee D.Y., Singh A. (2008). Elevated CRAF as a Potential Mechanism of Acquired Resistance to BRAF Inhibition in Melanoma. Cancer Res..

[26] Arozarena I., Wellbrock C. (2017). Overcoming resistance to BRAF inhibitors. Ann. Transl. Med..

[27] Raaijmakers M.I.G., Widmer D.S., Narechania A., Eichhoff O., Freiberger S.N., Wenzina J., Cheng P.F., Mihic-Probst D., DeSalle R., Dummer R. (2016). Co-existence of BRAF and NRAS driver mutations in the same melanoma cells results in heterogeneity of targeted therapy resistance. Oncotarget.

[28] Thomas N.E., Edmiston S.N., Alexander A., Millikan R.C., Groben P.A., Hao H., Tolbert D., Berwick M., Busam K., Begg C.B. (2007). Number of Nevi and Early-Life Ambient UV Exposure Are Associated with BRAF-Mutant Melanoma. Cancer Epidemiol. Biomark. Prev..

[29] Network N.C.C. Cutaneous Melanoma. https://www.nccn.org/professionals/physician_gls/pdf/cutaneous_melanoma_blocks.pdf.

[30] Kim S.Y., Hahn H.J., Lee Y.W., Choe Y.B., Ahn K.J., Kim S.N. (2015). Metaanalysis of BRAF mutations and clinicopathologic characteristics in primary melanoma. J. Am. Acad. Dermatol..

[31] Bombonato C., Ribero S., Pozzobon F., Puig-Butille J., Badenas C., Carrera C., Malvehy J., Moscarella E., Lallas A., Piana S. (2016). Association between dermoscopic and reflectance confocal microscopy features of cutaneous melanoma with BRAF mutational status. J. Eur. Acad. Dermatol. Venereol..

[32] Liu W., Kelly J.W., Trivett M., Murray W.K., Dowling J.P., Wolfe R., Mason G., Magee J., Angel C., Dobrovic A. (2007). Distinct Clinical and Pathological Features Are Associated with the BRAFT1799A(V600E) Mutation in Primary Melanoma. J. Investig. Dermatol..

[33] Armengot-Carbó M., Nagore E., García-Casado Z., Botella-Estrada R. (2018). The association between dermoscopic features and BRAF mutational status in cutaneous melanoma: Significance of the blue-white veil. J. Am. Acad. Dermatol..

[34] Pozzobon F., Puig-Butillé J., González-Alvarez T., Carrera C., Aguilera P., Alos L., Badenas C., Grichnik J., Malvehy J., Puig S. (2014). Dermoscopic criteria associated with BRAF and NRAS mutation status in primary cutaneous melanoma. Br. J. Dermatol..

[35] Mann G.J., Pupo G.M., Campain A.E., Carter C.D., Schramm S.-J., Pianova S., Gerega S.K., De Silva C., Lai K., Wilmott J.S. (2013). BRAF Mutation, NRAS Mutation, and the Absence of an Immune-Related Expressed Gene Profile Predict Poor Outcome in Patients with Stage III Melanoma. J. Investig. Dermatol..

[36] Hugdahl E., Kalvenes M.B., Puntervoll H.E., Ladstein R.G., Akslen L.A. (2016). BRAF-V600E expression in primary nodular melanoma is associated with aggressive tumour features and reduced survival. Br. J. Cancer.

[37] Tarhini A., Kudchadkar R.R. (2018). Predictive and on-treatment monitoring biomarkers in advanced melanoma: Moving toward personalized medicine. Cancer Treat. Rev..

[38] Ascierto P.A., Dummer R., Gogas H.J., Flaherty K.T., Arance A., Mandala M., Liszkay G., Garbe C., Schadendorf D., Krajsova I. (2020). Update on tolerability and overall survival in COLUMBUS: Landmark analysis of a randomised phase 3 trial of encorafenib plus binimetinib vs vemurafenib or encorafenib in patients with BRAF V600–mutant melanoma. Eur. J. Cancer.

[39] Coit D.G., Thompson J.A., Albertini M.R., Barker C., Carson W.E., Contreras C., Daniels G.A., DiMaio D., Fields R.C., Fleming M.D. (2019). Cutaneous Melanoma, Version 2.2019, NCCN Clinical Practice Guidelines in Oncology. J. Natl. Compr. Cancer Netw..

[40] Bhatia P., Friedlander P., Zakaria E.A., Kandil E. (2015). Impact of BRAF mutation status in the prognosis of cutaneous melanoma: An area of ongoing research. Ann. Transl. Med..

[41] Gershenwald J.E., Scolyer R.A., Hess K.R., Sondak V.K., Long G.V., Ross M.I., Lazar A.J., Faries M.B., Kirkwood J.M., McArthur G.A. (2017). Melanoma staging: Evidence-based changes in the American Joint Committee on Cancer eighth edition cancer staging manual. CA Cancer J. Clin..

[42] Ellerhorst J.A., Greene V.R., Ekmekcioglu S., Warneke C.L., Johnson M.M., Cooke C.P., Wang L.-E., Prieto V.G., Gershenwald J.E., Wei Q. (2011). Clinical Correlates of NRAS and BRAF Mutations in Primary Human Melanoma. Clin. Cancer Res..

[43] García-Casado Z., Traves V., Banuls J., Niveiro M., Gimeno-Carpio E., Jimenez-Sanchez A., Moragon M., Onrubia J., Oliver V., Kumar R. (2015). BRAF, NRAS and MC 1R status in a prospective series of primary cutaneous melanoma. Br. J. Dermatol..

[44] Anand K., Phung T.L., Bernicker E.H., Cagle P.T., Olsen R.J., Thomas J.S. (2020). Clinical Utility of Reflex Ordered Testing for Molecular Biomarkers in Lung Adenocarcinoma. Clin. Lung Cancer.

[45] The Society of Gynecologic Oncology of Canada Why is Tumour Testing in Ovarian Cancer Needed in Canada? An Opinion Statement Developed by the National BRCA Collaborative. http://g-o-c.org/wp-content/uploads/2020/02/20BRCACollaborative_TumourTestinginCanada_FINAL_Jan30.pdf.

[46] Michielin O., Van Akkooi A.C.J., Ascierto P.A., Dummer R., Keilholz U., ESMO Guidelines Committee (2019). Cutaneous melanoma: ESMO Clinical Practice Guidelines for diagnosis, treatment and follow-up. Ann. Oncol..

[47] Miller R., Walker S., Shui I., Brandtmüller A., Cadwell K., Scherrer E. (2020). Epidemiology and survival outcomes in stages II and III cutaneous melanoma: A systematic review. Melanoma Manag..

[48] Tan S.Y., Najita J., Li X., Strazzulla L.C., Dunbar H., Lee M.-Y., Seery V.J., Buchbinder E.I., Tawa N.E., McDermott D.F. (2019). Clinicopathologic features correlated with paradoxical outcomes in stage IIC versus IIIA melanoma patients. Melanoma Res..

[49] Normanno N. BRAF in Melanoma: ESMO Biomarker Factsheet. https://oncologypro.esmo.org/education-library/factsheets-on-biomarkers/braf-in-melanoma.

[50] Cheng L., Lopez-Beltran A., Massari F., MacLennan G.T., Montironi R. (2018). Molecular testing for BRAF mutations to inform melanoma treatment decisions: A move toward precision medicine. Mod. Pathol..

[51] Manfredi L., Meyer N., Tournier E., Grand D., Uro-Coste E., Rochaix P., Brousset P., Lamant L. (2016). Highly Concordant Results Between Immunohistochemistry and Molecular Testing of Mutated V600E BRAF in Primary and Metastatic Melanoma. Acta Derm. Venereol..

[52] Ihle M.A., Fassunke J., König K., Grünewald I., Schlaak M., Kreuzberg N., Tietze L., Schildhaus H.-U., Büttner R., Merkelbach-Bruse S. (2014). Comparison of high resolution melting analysis, pyrosequencing, next generation sequencing and immunohistochemistry to conventional Sanger sequencing for the detection of p.V600E and non-p.V600E BRAFmutations. BMC Cancer.

[53] Anderson S., Bloom K.J., Vallera D.U., Rueschoff J., Meldrum C., Schilling R., Kovach B., Lee J.R.-J., Ochoa P., Langland R. (2012). Multisite Analytic Performance Studies of a Real-Time Polymerase Chain Reaction Assay for the Detection of BRAF V600E Mutations in Formalin-Fixed, Paraffin-Embedded Tissue Specimens of Malignant Melanoma. Arch. Pathol. Lab. Med..

[54] Colomba E., Hélias-Rodzewicz Z., Von Deimling A., Marin C., Terrones N., Pechaud D., Surel S., Côté J.-F., Peschaud F., Capper D. (2013). Detection of BRAF p.V600E Mutations in Melanomas. J. Mol. Diagn..

[55] Mancini I., Simi L., Salvianti F., Castiglione F., Sonnati G., Pinzani P. (2019). Simi Analytical Evaluation of an NGS Testing Method for Routine Molecular Diagnostics on Melanoma Formalin-Fixed, Paraffin-Embedded Tumor-Derived DNA. Diagnostics.

[56] Bisschop C., ter Elst A., Bosman L.J., Platteel I., Jalving M., Berg A.V.D., Diepstra A., van Hemel B., Diercks G.F., Hospers G.A. (2018). Rapid BRAF mutation tests in patients with advanced melanoma: Comparison of immunohistochemistry, Droplet Digital PCR, and the Idylla Mutation Platform. Melanoma Res..

[57] A Monzon F., Ogino S., Hammond M.E.H., Halling K.C., Bloom K.J., Nikiforova M.N. (2009). The role of KRAS mutation testing in the management of patients with metastatic colorectal cancer. Arch. Pathol. Lab. Med..

[58] Tsiatis A.C., Norris-Kirby A., Rich R.G., Hafez M.J., Gocke C.D., Eshleman J.R., Murphy K.M. (2010). Comparison of Sanger Sequencing, Pyrosequencing, and Melting Curve Analysis for the Detection of KRAS Mutations: Diagnostic and Clinical Implications. J. Mol. Diagn..

[59] Melchior L., Grauslund M., Bellosillo B., Montagut C., Torres E., Moragón E., Micalessi I., Frans J., Noten V., Bourgain C. (2015). Multi-center evaluation of the novel fully-automated PCR-based Idylla™ BRAF Mutation Test on formalin-fixed paraffin-embedded tissue of malignant melanoma. Exp. Mol. Pathol..

[60] Chen N., Wang Y.-Y., Chuai Z.-R., Huang J.-F., Wang Y.-X., Liu K., Zhang L.-Q., Yang Z., Shi D.-C., Liu Q. (2015). High-Resolution Melting Analysis for accurate detection of BRAF mutations: A systematic review and meta-analysis. Sci. Rep..

[61] Vanni I., Tanda E.T., Spagnolo F., Andreotti V., Bruno W., Ghiorzo P. (2020). The Current State of Molecular Testing in the BRAF-Mutated Melanoma Landscape. Front. Mol. Biosci..

[62] Qu K., Pan Q., Zhang X., Rodriguez L., Zhang K., Li H., Ho A., Sanders H., Sferruzza A., Cheng S.-M. (2013). Detection of BRAF V600 Mutations in Metastatic Melanoma. J. Mol. Diagn..

[63] Cheema P.K., Raphael S., El-Maraghi R., Li J., McClure R., Zibdawi L., Chan A., Victor J., Dolley A., Dziarmaga A. (2017). Rate of Egfr Mutation Testing for Patients with Nonsquamous Non-Small-Cell Lung Cancer with Implementation of Reflex Testing by Pathologists. Curr. Oncol..

[64] Barth A., Wanek L.A., Morton D.L. (1995). Prognostic factors in 1521 melanoma patients with distant metastases. J. Am. Coll. Surg..

[65] Ascierto P.A., Ferrucci P.F., Fisher R., Del Vecchio M., Atkinson V., Schmidt H., Schachter J., Queirolo P., Long G.V., Di Giacomo A.M. (2019). Dabrafenib, trametinib and pembrolizumab or placebo in BRAF-mutant melanoma. Nat. Med..

[66] Grob J.J., Garbe C., Ascierto P., Larkin J., Dummer R., Schadendorf D. (2018). Adjuvant melanoma therapy with new drugs: Should physicians continue to focus on metastatic disease or use it earlier in primary melanoma?. Lancet Oncol..

[67] Long G.V., Stroyakovskiy D., Gogas H., Levchenko E., de Braud F., Larkin J., Garbe C., Jouary T., Hauschild A., Grob J.-J. (2015). Dabrafenib and trametinib versus dabrafenib and placebo for Val600 BRAF-mutant melanoma: A multicentre, double-blind, phase 3 randomised controlled trial. Lancet.

[68] Larkin J., Ascierto P.A., Dréno B., Atkinson V., Liszkay G., Maio M., Mandalà M., Demidov L., Stroyakovskiy D., Thomas L. (2014). Combined Vemurafenib and Cobimetinib in BRAF-Mutated Melanoma. N. Engl. J. Med..

[69] Long G.V., Eroglu Z., Infante J., Patel S., Daud A., Johnson D.B., Gonzalez R., Kefford R., Hamid O., Schuchter L. (2018). Long-Term Outcomes in Patients With BRAF V600–Mutant Metastatic Melanoma Who Received Dabrafenib Combined With Trametinib. J. Clin. Oncol..

[70] El-Deiry W.S., Goldberg R.M., Lenz H., Shields A.F., Gibney G.T., Tan A.R., Brown J., Eisenberg B., Heath E.I., Phuphanich S. (2019). The current state of molecular testing in the treatment of patients with solid tumors, 2019. CA Cancer J. Clin..

[71] Natsuhara K.H., Losk K., King T.A., Lin N.U., Camuso K., Golshan M., Pochebit S., Brock J.E., Bunnell C.A., Freedman R.A. (2018). Impact of Genomic Assay Testing and Clinical Factors on Chemotherapy Use After Implementation of Standardized Testing Criteria. Oncologist.

[72] Katz G., Romano O., Foa C., Vataire A.-L., Chantelard J.-V., Hervé R., Barletta H., Durieux A., Martin J.-P., Salmon R. (2015). Economic Impact of Gene Expression Profiling in Patients with Early-Stage Breast Cancer in France. PLoS ONE.

[73] Losk K., Freedman R.A., Lin N.U., Golshan M., Pochebit S.M., Lester S.C., Natsuhara K., Camuso K., King T.A., Bunnell C.A. (2017). Implementation of Surgeon-Initiated Gene Expression Profile Testing (Oncotype DX) Among Patients With Early-Stage Breast Cancer to Reduce Delays in Chemotherapy Initiation. J. Oncol. Pract..

[74] Aubin F., Gill S., Burkes R., Colwell B., Kamel–Reid S., Koski S., Pollett A., Samson B., Tehfe M., Wong R. (2011). Canadian Expert Group Consensus Recommendations: KRAS Testing in Colorectal Cancer. Curr. Oncol..

[75] Giri V. New Recommendations Offer Guidance for Clinicians and Patients on Genetic Testing for Prostate Cancer. https://ascopost.com/issues/july-10-2020/new-recommendations-offer-guidance-for-clinicians-and-patients-on-genetic-testing-for-prostate-cancer/.

[76] Laboratories M.C. BCR/ABL1 Qualitative Diagnostic Assay with Reflex to BCR/ABL1 p190 Quantitative Assay or BCR/ABL1 p210 Quantitative Assay, Varies. https://www.mayocliniclabs.com/test-catalog/Overview/65248.

[77] Craig J.W., Hasserjian R.P., Kim A.S., Aster J.C., Pinkus G.S., Hornick J.L., Steensma D.P., Lindsley R.C., DeAngelo D.J., Morgan E.A. (2020). Detection of the KITD816V mutation in myelodysplastic and/or myeloproliferative neoplasms and acute myeloid leukemia with myelodysplasia-related changes predicts concurrent systemic mastocytosis. Mod. Pathol..

[78] Assasi N., Blackhouse G., Campbell K., Weeks L., Levine M. (2015). Mismatch Repair Deficiency Testing for Patients with Colorectal Cancer: A Clinical and Cost-Effectiveness Evaluation.

[79] Mills A.M., Liou S., Ford J.M., Berek J.S., Pai R.K., Longacre T.A. (2014). Lynch Syndrome Screening Should Be Considered for All Patients With Newly Diagnosed Endometrial Cancer. Am. J. Surg. Pathol..

[80] Lewis J.S., Beadle B., Bishop J.A., Chernock R.D., Colasacco C., Lacchetti C., Moncur J.T., Rocco J.W., Schwartz M.R., Seethala R.R. (2017). Human Papillomavirus Testing in Head and Neck Carcinomas: Guideline From the College of American Pathologists. Arch. Pathol. Lab. Med..

[81] Smith A.L., Wong C., Cuggia A., Borgida A., Holter S., Hall A., Connor A.A., Bascuñana C., Asselah J., Bouganim N. (2018). Reflex Testing for Germline BRCA1, BRCA2, PALB2, and ATM Mutations in Pancreatic Cancer: Mutation Prevalence and Clinical Outcomes From Two Canadian Research Registries. JCO Precis. Oncol..

[82] Dummer R., Hauschild A., Lindenblatt N., Pentheroudakis G., Keilholz U. (2015). Cutaneous melanoma: ESMO Clinical Practice Guidelines for diagnosis, treatment and follow-up. Ann. Oncol..

