# Supplementary material for: Defining the Criteria for Reflex Testing for BRAF Mutations in Cutaneous Melanoma Patients
Source: Cancers (Basel). 2021 May 10;13(9):2282. doi: 10.3390/cancers13092282 (PMC8126223; doi:10.3390/cancers13092282)
Supplement: Supplementary file 1 [file cancers-13-02282-s001.zip › cancers-1189055-supplementary.pdf]

SUPPLEMENTARY MATERIALS

Table S1. Simplified overview of the pathologic staging of melanoma (American Joint Committee on Cancer 8th edition).

Stage		Tumor	Nodes	Metastases
**0**		Melanoma in situ	None	None
I	A	≤ 1mm, +/- ulceration	None	None
I	B	1 to 2mm, no ulceration	None	None
II	A	1 to 2 mm, + ulceration<br>2 to 4mm, no ulceration	None	None
II	B	2 to 4mm, + ulceration<br>>4mm, no ulceration	None	None
II	C	>4mm, + ulceration	None	None
III	A	Up to 1mm +/- ulceration<br>1 to 2mm, no ulceration	1 to 3 clinically occult nodes	None
III	B	No evidence of primary	1 clinically detected node, OR evidence of ILD *	None
		Up to 2mm +/– ulceration<br>2 to 4 mm, no ulceration	Up to 3 tumor-involved nodes, OR evidence of ILD	
III	C	No evidence of primary	>2 tumor-involved nodes, OR ILD reaching nearby nodes, OR matted nodes	None
		Up to 2mm +/- ulceration OR 2 to 4mm, no ulceration	≥ 4 tumor-involved nodes, OR ILD reaching nearby nodes, OR ≥ 1 matted node	
		2 to 4mm +/- ulceration OR > 4.0mm, no ulceration	≥ 1 tumor-involved node, +/- evidence of ILD	
		> 4.0mm, + ulceration	1 to 3 tumor-involved nodes OR evidence of ILD	
III	D	> 4.0mm, + ulceration	≥ 4 tumor-involved nodes<br>ILD reaching ≥ 2 nearby nodes<br>≥ 1 matted node	None
IV		Any	Any	Evidence of distant metastases

**ILD:* Intralymphatic disease refers to in-transit, satellite and/or microsatellite metastases.

Table S2. Summary of targeted therapy trials in the treatment of unresected stage III or IV melanoma and resected melanoma. *NR:* not reported.

RESECTABLE MELANOMA						
**Trial Drugs compared N**	**Patients included**	**Treatment**	**Median follow-up (months)**	**Response rate (%)**	**Recurrence-free survival (%)**	**Overall survival (%)**
**COMBI-AD [1,2]**<br>Dabrafenib + Trametinib versus placebo	Completely resected BRAF+ stage IIIA melanoma with LN metastasis ≥1mm, stage IIIB melanoma, stage	Dabrafenib + Trametinib (n=438)	60 months	NA	88% at 1 year<br>67% at 2 years<br>58% at 3 years<br>55% at 4 years<br>52% at 5 years	97% at 1 year<br>91% at 2 years<br>86% at 3 years

N=870	IIIC melanoma (according to AJCC 7)				Median RFS NR (HR versus placebo = 0.51 [95%CI = 0.42-0.61])	
		Placebo (n=432)	58 months	NA	56% at 1 year<br>44% at 2 years<br>39% at 3 years<br>38% at 4 years<br>36% at 5 years<br><br>Median RFS = 16.6 months	94% at 1 year<br>83% at 2 years<br>77% at 3 years
**BRIM 8 [3]**<br><br>Vemurafenib versus placebo (N = 498)	Cohort 1: stage IIC-IIIB melanoma (according to AJCC 7)	Vemurafenib (n=157)	30.8 months	NA	DFS*:<br><br>84% at 1 year<br>72% at 2 years<br><br>Median = NR	93% at 2 years
		Placebo (n=157)		NA	66% at 1 year<br>57% at 2 years<br><br>Median 36.9 months	87% at 2 years
	Cohort 2: stage IIIC melanoma (according to AJCC 7)	Vemurafenib (n=93)	33.5 months	NA	DFS*:<br>79% at 1 year<br>46% at 2 years<br><br>Median DFS = 23.1 months (HR versus placebo = 0.80 [95% CI 0.54-1.18], p = 0.26)	84% at 2 years
		Placebo (n=91)		NA	58% at 1 year<br>48% at 2 years<br><br>Median DFS = 15.4 months	85% at 2 years
**UNRESECTED MELANOMA**						
**Trial Drugs compared N**	**Patients included**	**Treatment**	**Median follow-up (months)**	**Response rate (%)**	**Progression-free survival (months) [95% CI]**	**Overall survival (months) [95% CI]**
**CoBRIM [4]**<br><br>Cobimetinib + Vemurafenib versus Vemurafenib<br><br>N=495	BRAF+ melanoma, unresected stage IIIC or stage IV, previously untreated	Cobimetinib + Vemurafenib (n=247)	14.2<br><br>(18.5 for OS analysis)	ORR = 70 CR = 16	12.3 [9.5-13.4]<br><br>(HR versus Vemurafenib = 0.58 [95% CI = 0.46-0.72] p <0.0001)	75% at 1 year<br>48% at 2 years<br><br>Median = 22.3 [20.3-not estimable]<br><br>(HR versus Vemurafenib = 0.70 [95%CI =

						0.55-0.90], p = 0.005)
		Vemurafenib (n=248)		ORR = 50<br>CR = 10	7.2 [5.6-7.5]	64% at 1 year<br>38% at 2 years<br><br>Median = 17.4 [95% CI = 15.0-19.8]
**COLUMBUS [5]**<br><br>Encorafenib + Binimetinib versus Encorafenib or Vemurafenib monotherapy<br><br>N=577	BRAF+ melanoma, unresected stage IIIB, IIIC or IV, treatment naïve or had progressed after previous first-line immunotherapy,	Encorafenib + Binimetinib (n=192)	32.1 (PFS)<br>48.8 (OS)	ORR = 64<br>CR = 13	56% at 1 year<br>37% at 2 years<br>29% at 3 years<br><br>Median = 14.9 [11.0-20.2] (HR versus Vemurafenib = 0.51 [95% CI = 0.39-0.67])	76% at 1 year<br>58% at 2 years<br>47% at 3 years<br><br>Median = 33.6 [24.4-39.2] (HR versus Vemurafenib = 0.61 [95% CI = 0.48-0.79])
		Vemurafenib (n=191)		ORR = 41<br>CR = 8	32% at 1 year<br>20% at 2 years<br>14% at 3 years<br><br>Median = 7.3 [5.6-7.9]	63% at 1 year<br>43% at 2 years<br>31% at 3 years<br><br>Median = 16.9 [14.0-24.5]
		Encorafenib (n=194)		ORR = 52<br>CR = 7	Median = 9.6 [7.4-14.8]	Median = 23.5 [19.6-33.6]
**COMBI-d [6]**<br><br>Dabrafenib + Trametinib versus Dabrafenib monotherapy<br><br>N=423	BRAF+ melanoma, unresected stage IIIC or stage IV melanoma, no prior systemic therapy	Dabrafenib + Trametinib (n=211)	20 (time on study)	ORR = 69<br>CR = 16%	Median 11.0 [8.0-13.9]<br><br>(HR versus Dabrafenib = 0.67 [95% CI = 0.53-0.84], p = 0.0004)	74% at 1 year<br>51% at 2 years<br><br>Median = 25.1 [19.2-not reached] (HR versus Dabrafenib = 0.71 [95% CI = 0.55-0.92], p = 0.0107)
		Dabrafenib (n=212)	16 (time on study)	ORR = 53%<br>CR = 13%	Median = 8.8 [5.9-9.3]	68% at 1 year<br>42% at 2 years<br><br>Median = 18.7 [15.2-23.7]
**COMBI-v [7]**<br><br>Dabrafenib + Trametinib versus Vemurafenib monotherapy<br><br>N=704	BRAF+ melanoma, unresected stage IIIC or stage IV melanoma, no prior systemic therapy	Dabrafenib + Trametinib (n=352)	11	ORR = 64<br>CR = 13%	Median = 11.4<br><br>(HR versus Vemurafenib = 0.56 [95% CI = 0.46-0.69], p <0.001)	72% at 1 year<br><br>Median = not reached (HR versus Vemurafenib = 0.69 [95% CI = 0.53-0.89], p = 0.005)
		Vemurafenib (n=352)	10	ORR = 51<br>CR = 8%	Median = 7.3	65% at 1 year<br><br>Median = 17.2
**COMBI-d and COMBI-v**	BRAF+ melanoma, unresected stage IIIC or stage IV melanoma,	Dabrafenib +	22	ORR = 68%	31% at 2 years<br>24% at 3 years<br>21% at 4 years	52% at 2 years<br>44% at 3 years<br>37% at 4 years

**(pooled analysis) [8]**	no prior systemic therapy	Trametinib (n=563)	CR = 19%	19% at 5 years<br>Median = 11.1	34% at 5 years<br>Median = 25.9 months

Because of the prespecified hierarchical testing of cohort 2 before cohort 1, and because the primary endpoint was not met in cohort 2, the analyses in cohort 1 cannot be regarded as significant.

Overall Response Rate (ORR) = overall response rate; CR = complete response rate; Duration Free Survival (DFS) measured in BRIM8.

References:

1. Long GV et al. Adjuvant Dabrafenib plus Trametinib in Stage III BRAF-Mutated Melanoma. NEJM. 2017. 377: 1813-1823.
2. Dummer R et al. Five-Year Analysis of Adjuvant Dabrafenib + Trametinib in Stage III Melanoma. NEJM. 2020. 383: 1139-1148.
3. Maio M et al. Adjuvant vemurafenib in resected, BRAFv600 mutation-positive melanoma (BRIM8) : a randomized, double-blind, placebo-controlled, multicentre, phase 3 trial. Lancet Oncology. 2018. 19: 510-520.
4. Ascierto PA et al. Cobimetinib combined with vemurafenib in advanced BRAFv600-mutant melanoma (coBRIM) : updated efficacy results from a randomized, double-blind, phase 3 trial. Lancet Oncology. 2016. 17: 1248-1260.
5. Ascierto PA et al. Update on tolerability and overall survival in COLUMBUS: landmark analysis of a randomized phase 3 trial of encorafenib plus binimetinib vs vémurafénib or encorafenib in patients with BRAF V600-mutant melanoma. European Journal of Cancer. 2020. 126: 33-44.
6. Long GV et al. Dabrafenib plus trametinib versus dabrafenib and placebo for Val600 BRAF-mutant melanoma: a multicenter, double-blind, phase 3 randomised controlled trial. The Lancet. 2016. 386 (9992): 444-451.
7. Robert C. et al. Improved Overall Survival in Melanoma with Combined Dabrafenib + Trametinib. NEJM. 2015. 372: 30-39.
8. Robert C et al. Five-year Outcomes with Dabrafnib plus Trametinib in Metastatic Melanoma. NEJM. 2019. 381: 626-636.
